# The use of antenatal corticosteroids in preterm labour for the prevention of perinatal mortality in hospitals in Tanzania

**DOI:** 10.4314/ahs.v24i1.18

**Published:** 2024-03

**Authors:** Stanley Mwita

**Affiliations:** Department of Pharmaceutics and Pharmacy Practice, Catholic University of Health and Allied Sciences, Mwanza, Tanzania

**Keywords:** Antenatal corticosteroids, preterm labour, perinatal mortality

## Abstract

**Background:**

Antenatal corticosteroids (ACS) are given to pregnant women at risk of preterm delivery to hasten the maturation of the lungs, lowering the risk of newborn respiratory distress syndrome (RDS) and perinatal mortality.

**Objective:**

The aim of this study was to determine whether exposure to ACS was associated with lower rates of perinatal mortality and RDS in preterm infants delivered by women with preterm labour.

**Methods:**

This is a secondary analysis of data from four hospitals in Mwanza, Tanzania. All singletons and twins born to women who were in preterm labour between July 2019 and February 2020 and delivered in-hospital between 24 and 34 weeks of gestation were included. Data were recorded from participants' medical records and analysed using STATA Version 14.

**Results:**

Over an eight-month period, 588 preterm infants were delivered to 527 women. One hundred and ninety (36.1%) women were given ACS. Infants who were exposed to ACS in utero had a lower rate of perinatal mortality (6.8% vs 19.1%) and RDS (12.3% vs 25.9%) compared to those not exposed to ACS. In adjusted multivariable models, ACS exposure was related to a lower risk of perinatal mortality, aRR 0.23 (95% CI 0.13 – 0.39), and RDS, aRR 0.45 (95% CI 0.30 – 0.68).

**Conclusion:**

ACS significantly reduced the risk of perinatal mortality and RDS among preterm infants exposed to ACS in utero and delivered by women in preterm labour. The use of ACS should be encouraged in low-resource settings where preterm birth is prevalent to improve perinatal outcomes.

## Introduction

Preterm birth is defined by the World Health Organization (WHO) as a live birth before 37 weeks of gestation.^1^ Preterm birth accounts for 35% of neonatal deaths worldwide, with an estimated 14.9 million preterm births per year. Worldwide, preterm birth is the main cause of perinatal mortality. 2 Stillbirths and neonatal deaths within the first week of life are referred to as perinatal mortality.^3^ Preterm birth is preceded by preterm labour in roughly 70% of all cases.^4^ In a Tanzanian study, 478 (56.6%) women with singleton pregnancies who delivered prematurely had preterm labour.^5^

Respiratory distress syndrome (RDS) is the most common cause of perinatal mortality and is a significant consequence of preterm birth. 6,7 RDS is caused by the absence or insufficient production of pulmonary surfactant and the associated immaturity of the lungs.^8^ Antenatal corticosteroids (ACS) are medicines given to pregnant women who are at risk of giving birth prematurely. When given to a fetus, these steroids hasten the maturation of the lungs, lowering the risk of newborn RDS and perinatal mortality.^9^,^10^ Dexamethasone and betamethasone are corticosteroids used for this purpose. Dexamethasone, on the other hand, has an advantage over betamethasone in terms of cost and availability, and it is currently listed on the WHO essential medicine list for use in pregnant women.^11^

According to research articles included in a previous scoping review, the use of ACS in hospitals with high levels of newborn care in low- and middle-income countries (LMICs) is associated with lower rates of neonatal mortality and RDS. However, no benefit was seen in health facilities with limited resources.^12^ Despite the fact that the benefit of ACS varies depending on the delivery indications (e.g., antepartum hemorrhage, pre-eclampsia or eclampsia, premature preterm rupture of membrane (PPROM), preterm labour, etc.), most prior research did not assess its impact on perinatal outcomes.^13^-^17^ Furthermore, a secondary analysis of a retrospective medical records study of 36 women with severe pre-eclampsia and eclampsia conducted in Ndala Hospital, Tanzania between July 2011 and December 2012 reported poor perinatal outcomes among infants who were exposed to ACS.^18^ The existing evidence suggests that ACS can help women with PPROM reduce perinatal morbidity and mortality. 19,20 However, there is a scarcity of research that has been conducted among women with preterm labour. Thus, this study investigated if exposure to ACS was associated with lower rates of perinatal mortality and RDS in preterm infants delivered by women with preterm labour.

## Methods

### Study design and setting

This is a secondary analysis of data from an observational prospective chart review study that examined whether ACS exposure was associated with lower perinatal mortality in preterm infants in the general population. 13 It took place in four hospitals in the districts of Nyamagana and Sengerema, which are two of the seven districts in the Mwanza region of northwest Tanzania. The facilities that took part were: Bugando Medical Centre (a tertiary consultant zonal referral hospital), Sekou Toure Regional Referral Hospital, Nyamagana District Hospital, and Sengerema District Designated Hospital. These hospitals serve a substantial percentage of the population in Tanzania's Lake Zone, providing obstetric and neonatal care.

### Sampling and data collection procedures

All singletons and twins born between July 2019 and February 2020 who met the following criteria were included in the study: (1) infants of a mother who was in preterm labour; (2) infants delivered in-hospital between 24 weeks and 34 weeks of gestation; and (3) infants born within 7 days of the mother receiving ACS. Infants with a congenital abnormality as documented in the medical record were excluded. The key predictor variable was ACS (dexamethasone) exposure, which was given in four doses of 6 mg of dexamethasone every 12 hours as per the standard ACS protocol. The women in the study were divided into two groups: the No-ACS group and the ACS group (women who had been given at least one dose).^21^ The primary outcome was perinatal mortality, while the secondary outcome was a diagnosis of RDS.

The lead investigator and trained research assistants who were enrolled or registered nurses working in the hospital's labour wards and neonatal units analyzed the medical records of women and their infants. Data on all pregnant women who were admitted to the hospital between 24 0/7 weeks and 34 6/7 weeks of pregnancy was collected to control for selection bias. Information on whether the mother was exposed to ACS or not, perinatal mortality, and the infant's RDS status for each participant were recorded. The women's and infants' medical records also provided maternal age, gestational age (weeks), parity, marital status, antenatal care visits (days), mode of delivery, level of health facility, pregnancy status (singleton/twin), birth weight (grams), and neonate sex. Women's gestational ages were measured using their last regular menstrual period, fundal height, and/or ultrasound results.

### Data analysis

The data was analysed using STATA Version 14. To detect differences in categorical variables between the ACS and No ACS groups, chi-square tests or Fisher exact tests were used. The Mann-Whitney U tests were employed to see if there were any variations in maternal age, gestational age, or birth weight between groups. Cross-tabulation and Chi-square testing were used to check for differences in perinatal mortality and RDS between the ACS and No ACS groups. Modified Poisson regressions were used to investigate the associations between ACS exposure and perinatal outcomes. To account for the clustering effect and non-independence of twins, the model was fitted by utilizing generalized estimating equations (GEE). The effects of ACS on perinatal mortality and RDS were investigated using multivariate analyses that controlled for gestational age, birth weight, level of health facility, and mode of delivery. P values of less than 0.05 were considered statistically significant. Data are presented as frequencies (percentages), median (IQR) and relative risks (RR) with 95% CIs as appropriate.

### Ethical consideration

The Joint Ethics and Research Review Committee of the Catholic University of Health and Allied Sciences and Bugando Medical Center granted the ethical approval (IRB approval number: CREC/368/2019). Secondary data was gathered from health records. No patients were contacted for this study. The ethics committee did not require informed consent from participants.

## Results

Over an eight-month period, 588 preterm infants were delivered to 527 women. The median (IQR) maternal age was 26 (22 - 30), and the median (IQR) gestational age was 32 (30 - 34). Almost a third of the women were nulliparous; 86.3% were married; 40.4% had at least four antenatal care visits; 27.9% were delivered via cesarean section; and 36.1% were given ACS. Furthermore, more than one-third of women gave birth in a tertiary care facility. About 20.4% of infants were twins. Infants' median (IQR) birthweight was 1900 (1500–2400), and almost half were males (Table 1).

**Table 1:** Maternal and infants' demographic characteristics

Maternal (n=527)	Median/Frequency	IQR/Percentage
Maternal age	26	22-30
Gestational age	32	30-34
Nulliparity	163	30.9
Married	455	86.3
More than 3 Antenatal care visits†	206	40.4
Cesarean section delivery	147	27.9
ACS given	190	36.1
**Level of health facility**		
Tertiary zonal hospital	202	38.3
Regional hospital	175	33.2
District hospital	150	28.5
**Infants (n=588)**		
Twins	120	20.4
Birthweight	1900	1500-2400
Males	303	51.5

† Denominator included only those who attended antenatal care (i.e., 510)

No significant differences in median maternal age, parity, groups differ on the following variables: gestational age, marital status, parity, birthweight, or gender were found days of antenatal care attended, mode of delivery, health between the ACS and No ACS groups. However, the facility level, and pregnancy status (Table 2).

**Table 2:** Maternal and infants baseline characteristics by ACS treatment

Maternal	ACS given (n=190)	No ACS (n=337)	P Values
Maternal age, M (IQR)	26 (22-31)	26 (22-30)	0.720
Gestational age	32 (29-34)	33 (31-34)	<0.001
Nulliparity, N (%)	65 (34.2)	98 (29.1)	0.221
Married	165 (86.8)	290 (86.1)	0.800
More than 3 Antenatal care visits†	93 (49.5)	113 (35.1)	0.001
Cesarean section delivery	75 (39.5)	72 (21.4)	<0.001
**Level of health facility**			
Tertiary zonal hospital	137 (72.1)	65 (19.3)	<0.001
Regional hospital	43 (22.6)	132 (39.2)	
District hospital	10 (5.3)	140 (41.5)	
Infants	**ACS given (n=206)**	**No ACS (n=382)**	
Twins	30 (14.6)	90 (23.6)	0.013
Birthweight	1900 (1500-2400)	2000 (1700-2300)	0.119
Males	106 (51.4)	197 (51.5)	0.950

† Denominator included only those who attended antenatal care (ACS 188, No ACS 322)

Unadjusted estimates of perinatal outcomes are presented in Table 3. Infants who were exposed to ACS in utero had a lower rate of perinatal mortality (6.8% vs 19.1%) and RDS (12.3% vs 25.9%) compared to those not exposed to ACS.

**Table 3:** Perinatal outcomes for the ACS and No ACS groups

	ACS	No-ACS	P-value
**Outcomes**			
Perinatal Mortality	14/206 (6.8%)	73/382 (19.1%)	<0.001
RDS	25/203 (12.3%)	88/340 (25.9%)	<0.001

In adjusted multivariable models, ACS exposure was related to a lower risk of perinatal mortality, aRR 0.23 (95% CI 0.13-0.39), and RDS, aRR 0.45 (95% CI 0.30-0.68). (Table 4).

**Table 4:** Univariate and multivariate analysis of the associations between ACS exposure and perinatal outcomes

Outcomes	*Crude relative risk (95%CI)	*Adjusted relative risk (95%CI)
Perinatal mortality	0.36 (0.21 – 0.62)	0.23 (0.13-0.39)
RDS	0.49 (0.32 – 0.74)	0.45 (0.30-0.68)

* Model adjusted for gestational age, birthweight, level of health facility and mode of delivery

## Discussion

ACS administered to women at risk of premature delivery enhances the chances of their babies breathing and surviving. The aim of this study was to determine whether exposure to ACS was associated with lower rates of perinatal mortality and RDS in preterm infants delivered by women with preterm labour in hospitals located in Mwanza, Tanzania. Preterm infants exposed to ACS in utero and delivered by women in preterm labour had a lower rate of perinatal mortality and RDS than those who were not exposed to ACS and were less likely to have both perinatal mortality and RDS. These findings are similar to those in the parent study, which included the entire study population regardless of delivery indications. 13 However, the adjusted relative risk reported in this study is lower compared to that of the general population, i.e., 0.23 vs 0.34 and 0.45 vs 0.58 for perinatal mortality and RDS, respectively. This indicates that infants born to mothers with preterm labur are more likely to benefit from ACS administration than the general population. Previous studies demonstrated that the administration of ACS to women with pre-eclampsia or eclampsia, antepartum hemorrhage, and PPROM without chorioamnionitis resulted in a beneficial effect on reducing the risks of neonatal mortality and morbidities. 19,22-25

The results, however, did not support the ACT trial's findings, which found no difference in perinatal mortality between the ACS-exposed and unexposed groups. The ACT trial was a study carried out in rural and semi-urban clusters within six countries (i.e., Argentina, Guatemala, India, Kenya, Pakistan, and Zambia). 15 This may be due to variations in baseline characteristics of the mother and the fetus as well as variations in health care settings. The ACT trial provided information on the delivery of care at all levels, including primary healthcare and community-based care. In contrast, only hospital deliveries at district, regional, and zonal hospitals were included in the current study. The fact that these hospitals offer better care than primary healthcare facilities may have contributed to the positive findings of our study. Notably, the WHO only advises the use of ACS in cases where the gestational age is known, preterm delivery is about to occur, and the delivery will take place in a setting that can provide for both the mother and the baby. 11 Moreover, the ACT trial did not explicitly consider indications for preterm delivery (e.g., preterm labour, pre-eclampsia, PPROM, and antepartum hemorrhage) on perinatal mortality, while the current study included women with preterm labour only. The previous systematic review supported the use of ACS in women at risk of preterm birth in low, medium, and high-resource settings. Treatment with ACS has been reported to reduce the risk of perinatal death, neonatal death, and RDS, particularly in settings with advanced neonatal care. 26 Despite the benefits of ACS reported in the current study, it should be noted that only 36.1% of women with preterm labour received the therapy. The ACS operational guidelines must be disseminated and put into practice. Preterm birth care needs to be standardized and under stronger supervision in hospitals. 27 Facilities need to be more prepared to meet the requirements for ACS use, especially when it comes to preterm labour. However, increasing the use of ACS without supporting it with adequate quality maternal and newborn care might jeopardize improvements in preterm birth outcomes. 27

## Strength and limitation

The strength of this study was the inclusion of women with only one indication of preterm birth, i.e., women with preterm labour. This study in Tanzania is the first to document the benefits of ACS administration for infants delivered to mothers who experienced preterm labour. However, the observational study design used has some limitations. As a result, the findings may have been influenced by the biases inherent to observational studies (such as selection bias, information bias, and confounding). Selection bias was controlled by including all stillbirths and live births of preterm infants delivered at the selected hospitals between 24 weeks 0 days and 34 weeks 6 days of gestation between July 2019 and February 2020. Informational bias was controlled for by using a standard data collection form and confounding by considering a number of factors that could be associated with exposure to ACS and pregnancy outcomes.

## Conclusion

ACS significantly reduced the risk of perinatal mortality and RDS among preterm infants exposed to ACS in utero and delivered by women in preterm labour. The use of ACS should be encouraged in low-resource settings where preterm birth is prevalent to improve perinatal outcomes.

## Abbreviations

ACS: Antenatal corticosteroids
LMICs: Low- and middle-income countries
PPROM: Premature preterm rupture of membrane
RDS: Respiratory distress syndrome
WHO: World Health Organization
